# MicroRNA Array Normalization: An Evaluation Using a Randomized Dataset as the Benchmark

**DOI:** 10.1371/journal.pone.0098879

**Published:** 2014-06-06

**Authors:** Li-Xuan Qin, Qin Zhou

**Affiliations:** Department of Epidemiology and Biostatistics, Memorial Sloan Kettering Cancer Center, New York, New York, United States of America; National Institute of Environmental and Health Sciences, United States of America

## Abstract

MicroRNA arrays possess a number of unique data features that challenge the assumption key to many normalization methods. We assessed the performance of existing normalization methods using two microRNA array datasets derived from the same set of tumor samples: one dataset was generated using a blocked randomization design when assigning arrays to samples and hence was free of confounding array effects; the second dataset was generated without blocking or randomization and exhibited array effects. The randomized dataset was assessed for differential expression between two tumor groups and treated as the benchmark. The non-randomized dataset was assessed for differential expression after normalization and compared against the benchmark. Normalization improved the true positive rate significantly in the non-randomized data but still possessed a false discovery rate as high as 50%. Adding a batch adjustment step before normalization further reduced the number of false positive markers while maintaining a similar number of true positive markers, which resulted in a false discovery rate of 32% to 48%, depending on the specific normalization method. We concluded the paper with some insights on possible causes of false discoveries to shed light on how to improve normalization for microRNA arrays.

## Introduction

Normalization is an essential step of microarray data preprocessing [1]. It aims to remove non-biologic effects resulting from the experimental process so that biologic effects can be accurately identified [2], [3]. Methods for array normalization have been developed in the context of mRNA expression arrays. Main-stay approaches, such as median normalization and quantile normalization, are based on the data of all genes on the array, which we call “all-gene” methods [4], [5]. They rely on the assumption that only a very small proportion of the molecular markers on the array are differentially expressed, or that the numbers of over- and under-expressed markers are similar.

MicroRNAs (miRNAs) are a prevalent class of small single-stranded non-coding RNAs that negatively regulate gene expression by inducing mRNA degradation and translational repression, and are involved in a wide variety of cellular functions such as proliferation, differentiation, and apoptosis [6]–[8]. MiRNA arrays possess a number of unique data characteristics comparing with mRNA arrays. First, miRNA arrays contain markers for a much smaller number of miRNAs – the Agilent miRNA arrays (release 16.0) have markers for about 1,300 miRNAs, while mRNA arrays typically have markers for tens of thousands of genes. Second, differential expression is more likely to be common and asymmetric among miRNAs. The majority of miRNAs are expected to express in a very tissue-specific manner [9]–[12]. They were found to be important in tumorigenesis and show widespread differential expression between malignant and normal cells caused by mechanisms such as chromosomal alterations, DNA point mutations, and epigenetic changes [11], [13]. As a result, miRNA array data challenge the assumption of the all-gene methods (that the proportion of differentially expressed markers is small or the amount of over- and under-expression is similar). Therefore, the performance of normalization methods needs to be re-assessed for miRNA arrays using genuine benchmark datasets that realistically represent miRNA array data characteristics.

In this paper, we report the results from an empirical evaluation of normalization methods using a pair of miRNA array datasets generated at Memorial Sloan Kettering Cancer Center [14]. This study examined miRNA expression in a set of 96 high-grade serous ovarian cancer samples and 96 endometrioid endometrial cancer samples, which were all newly diagnosed, previously untreated, and collected at Memorial Sloan Kettering Cancer Center between 2000 and 2012, using Human miRNA microarrays (Agilent Technologies). Two datasets were generated for the same set of tumor samples using different experimental designs: (1) in one dataset, arrays were assigned to samples using a blocked randomization design and handled by an experienced technician in one single run; (2) in another dataset, arrays were processed in the order that specimen were collected and handled by two technicians in multiple runs. The randomized dataset contained no confounding array effects and required no normalization, while the non-randomized dataset possessed array effects and needed normalization. The randomized dataset was assessed for differential expression between two tumor groups and treated as the benchmark. The non-randomized dataset was assessed for differential expression after normalization (with or without a separate batch adjustment step) and compared against the benchmark. We concluded the paper by providing insights on possible causes of false discoveries and potential directions to further improve normalization for microRNA arrays.

## Methods

The use of human tumor tissues in our study was approved by the Memorial Sloan Kettering Cancer Center Institutional Review Board.

### Justification for the randomized data as the benchmark

Analysis of variance (ANOVA) has been successfully used to model the relation between mRNA gene expression and sample group, which attributes gene expression variation to factors such as marker effect, sample effect, and stochastic noise [15]–[18]. Here we use ANOVA to model miRNA expression and thus obtain insights on how randomization removes confounding array effects.

Let 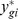 denote the true underlying expression level for marker g and sample i, and
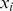
denote sample group (an indicator variable taking values 0 and 1 for a two-group study such as a case-control study). We can model 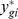 as the sum of marker effect 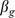, sample effect 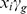, and stochastic noise 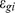.
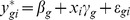



Let 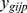 denote the observed expression level measured by microarray j for marker g, sample i, and replicate p. Agilent release 16.0 arrays contain 3,523 markers (that is, probes) representing 1,347 miRNAs and 10–40 replicates for each marker. Since j = 1 for all samples in the randomized data, we will use 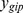 for simplification. 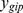
can be modeled as the sum of true expression level 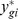, array effect 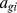, and measurement error 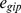. 
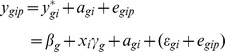



In the setting of marker-specific comparisons (that is, one between-group comparison per marker), we assume that (1) 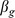 is a fixed effect representing the baseline expression for marker g, (2) 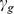
is a fixed effect representing the difference of expression between sample groups for marker g and is the parameter of interest, (3) 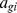 is a random effect whose mean depends on non-biologic factors such as array production batch, hybridization run, technician, and scanner, (4) 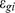
and 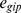 are random effects each normally distributed with mean 0, and (5) all the random effects are independent. Our model uses a most general form for array effects and allows it to be marker- and sample-specific. When a sample is profiled on only one array, array effect 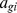 is not identifiable from sample effect 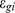. The goal of normalization hence is to introduce reasonable assumptions and effectively model array effects across markers so as to make array effects identifiable, estimable, and subsequently removable.

Different from normalization, randomization requires no modeling of array effects and it removes their confounding effect by balancing them between two sample groups. The mean of the observed expression 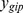 among control samples and case samples are denoted as 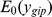 and 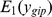, respectively, and they can be expressed as:
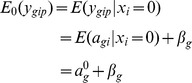


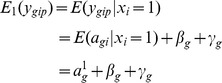



Hence the difference in the observed average expression between cases and controls is
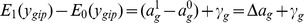



That is, the difference in observed means is biased by 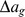.

The variance of the observed expression 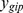 can be expressed as 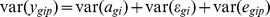



Thus the presence of array effects influences both the accuracy and precision of the estimate of 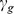, and consequently the accuracy of the hypothesis test for detecting markers that are truly differentially expressed. The estimated 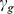, the parameter of interest, will achieve the best accuracy and precision when 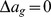 and 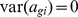.

The randomized dataset in our study included 192 arrays. Agilent miRNA arrays have an 8-plex block design, with eight arrays on each slide arranged as two rows and four columns. When assigning arrays to sample groups we used the blocked randomization design with row and column balance in order to ensure the best balance. There are six possible configurations where the numbers of cases and controls are equal on any row and any column of a slide [14]. We assigned the 24 slides for the 192 samples to one of the six configurations with equal probabilities and dedicated the arrays to one of the two sample groups. We then assigned each group of arrays to a random permutation of the samples in the corresponding group. As the result of randomization, 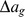 is close to zero for all markers in the randomized data.

We carefully planned our study for generating the randomized dataset. All 192 arrays were ordered from the same manufacture batch. Their hybridization and production were processed in one run by a single experienced technician at the Memorial Sloan Kettering Cancer Center Genomics Core Lab. As a result of the uniform array handling, 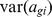 is minimal in the randomized data.

### Statistical analysis of the randomized data

#### Data preprocessing

We analyzed the data both with and without background subtraction, and found similar results in terms of the relative performance of normalization methods. We report the results for the analyses without background subtraction here. There was minimum variation among replicates for the same probe [14]. We summarized data from replicates for the same probe using the median on the log2 scale.

#### Differential expression analysis

We assessed evidence against the null hypothesis of equivalent expression, using the t statistic comparing two sample groups in the randomized data [19]. A separate test was performed for each of the 3,523 markers on the Agilent array and a two-sided p-value was calculated. The p-values were then used to derive a marker set at a given significance level: markers whose p-values were smaller than the significance level were declared differentially expressed, and those having larger p-values were declared non-differentially expressed.

### Statistical analysis of the non-randomized data

#### Data preprocessing

We preprocessed the non-randomized data with array normalization followed by probe summarization on the log2 scale. We applied commonly-used methods for normalization and used the same approach to probe summarization as for the randomized dataset. The normalization methods we used were median normalization, quantile normalization, cyclic loess normalization, and variance stabilizing normalization (Table S1). Briefly, median normalization shifts the data on an array by a constant so that arrays share the same median [20]; quantile normalization calculates a reference distribution as the averaged order statistics across arrays and then reset the order statistics on each array to this reference distribution [4]; cyclic loess normalization iterates through array pairs in a pre-specified order, and for each pair, it plots the difference of the two arrays versus their average intensity, fits a loess curve, and uses it as the new horizontal axis [4]; variance stabilizing normalization transforms the data (before log2 transformation) using a family of parametric transformations so that the variance of the resulted data is independent of the mean [21]. All four methods are based on the data of all markers on the array. In addition to normalization, we also tested whether adding a batch adjustment step before normalization can further improve the accuracy of biomarker discovery. We used two batch adjustment methods that have been proposed to adjust for gross differences between arrays: (1) standardization and (2) ComBat [22], [23].

#### Differential expression analysis

The preprocessed data were analyzed for differential expression using the same approach as that for the randomized dataset. The resulting p-values and the differential expression status based on the non-randomized data were compared with the differential expression status derived from the randomized data using ROC curves and cross tabulation, respectively [24].

## Results and Discussion

### Empirical evaluation of array normalization

**Figure 1:**
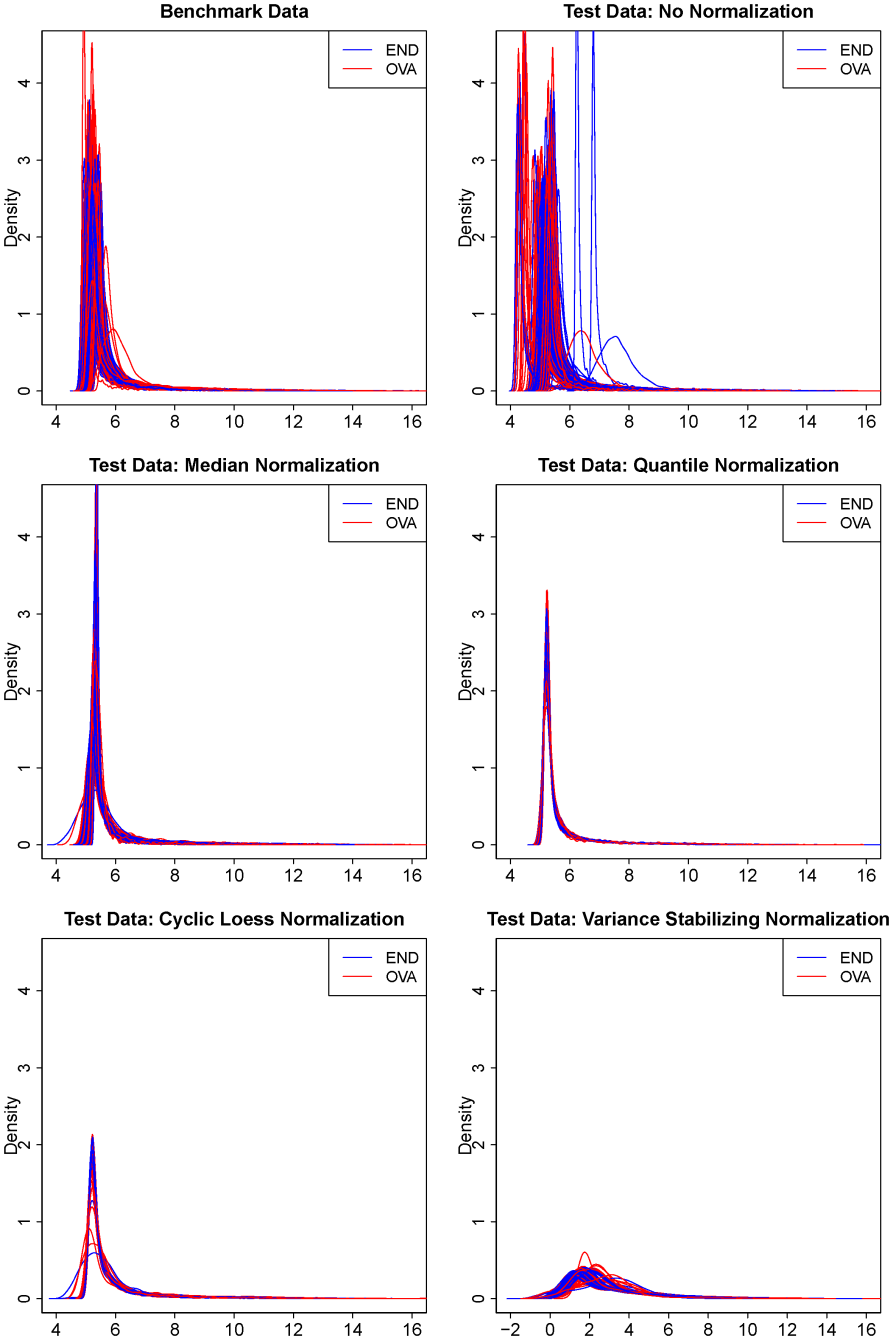
Density curves for the benchmark data and the test data with or without normalization. Each density curve represents the data for one array. Arrays for endometrial samples are colored in blue, and arrays for ovarian samples in red.

For the purpose of evaluating the effect of normalization on the accuracy of biomarker discovery, we called the randomized data as the benchmark data and the non-randomized data as the test data. Figure 1 shows the effect of normalization on the overall distribution of the test data. Table 1 shows the relative accuracy of biomarker detection in the normalized test data comparing with the benchmark data.

**Table 1:** Results of differential expression analysis of the test data before and after normalization, in comparison with the benchmark data.

	Number of Markers Claimed Positive	True Positive Rate	False Positive Rate	False Discovery Rate
**No normalization**	1934	0.53 (185/351)	0.55 (1749/3172)	0.90
**Median normalization**	639	0.85 (297/351)	0.11 (342/3172)	0.54
**Quantile normalization**	708	0.93 (328/351)	0.12 (380/3172)	0.54
**Cyclic loess normalization**	732	0.96 (336/351)	0.12 (396/3172)	0.54
**Variance stabilizing normalization**	723	0.89 (314/351)	0.13 (409/3172)	0.57

Among the 3,523 markers on the Agilent array, 351 markers (10%) were identified to be differentially expressed in the benchmark data, indicating a moderately abundant level of asymmetric differential expression. Without normalization, 1934 markers (55%) were identified to be differentially expressed at a p-value cutoff of 0.01 in the test data, which was associated with a true positive rate (TPR) of 185/351 (53%), a false positive rate (FPR) of 1749/3172 (55%), and a false discovery rate (FDR) of 90%. Almost all of the false positive markers had very low expression levels and some of the false negative markers had medium to high expression levels in the benchmark data (Figure S1).

With normalization, the number of differentially expressed markers was reduced to 639 (TPR: 85%, FPR: 11%, FDR: 54%) for median normalization, 708 (TPR: 93%, FPR: 12%, FDR: 54%) for quantile normalization, 732 (TPR: 96%, FPR: 12%, FDR: 54%) for cyclic loess normalization, and 723 (TPR: 89%, FPR: 13%, FDR: 57%) for variance stabilizing normalization. We also evaluated how accurately the test-data p-values ranked the markers using the ROC curve and observed similar results (Figure S2). Normalization improved the detection of differentially expressed markers with both an increased number of true positive markers and a decreased number of false positive markers in the empirical analysis of our data. However, even with the application of normalization, the number of false positive markers was still as many as the number of true positive markers, corresponding to a false discovery rate of about 50%, regardless of the specific normalization method used.

### Empirical evaluation of array normalization following batch adjustment

We next examined whether an addition of a batch adjustment step before array normalization can further improve the accuracy of differential expression detection. We compared the accuracy of calling differentially expressed markers with versus without batch adjustment, in combination with each normalization method tested in our study. It showed that normalization alone called highly similar markers positive to normalization combined with standardization, and moderately similar to normalization combined with ComBat (Figure S3).When comparing with the benchmark data, adding standardization to normalization made virtually no change to the number of false and true positive markers (Figure S4); adding ComBat further reduced the number of false positive markers while maintaining a similar number of true positive markers, which led to a FDR of 32% to 48% depending on the specific normalization method (Table 2 and Figure S4). Taken together, these results support the use of ComBat in combination with normalization to improve the accuracy of biomarker discovery in the analysis of miRNA array data. The choice of normalization method, however, depends on a trade-off of true positive rate and false positive rate. When combined with ComBat, quantile normalization (TPR: 93%, FPR: 9%, FDR: 48%) and cyclic loess normalization (TPR: 91%, FPR: 7%, FDR: 42%) had a high TPR but a relatively high FPR, while median normalization (TPR: 84%, FPR: 4%, FDR: 32%) and no normalization (TPR: 66%, FPR: 1%, FDR: 16%) had a relatively low TPR but a low FPR.

**Table 2:** Results of differential expression analysis of the test data before and after a combination of ComBat and normalization, in comparison with the benchmark data.

	Number of Markers Claimed Positive	True Positive Rate	False Positive Rate	False Discovery Rate
**No normalization**	275	0.66 (232/351)	0.01 (43/3172)	0.16 (43/275)
**Median normalization**	434	0.84 (296/351)	0.04 (138/3172)	0.32 (138/434)
**Quantile normalization**	623	0.93 (327/351)	0.09 (296/3172)	0.48 (296/623)
**Cyclic loess normalization**	545	0.91 (318/351)	0.07 (227/3172)	0.42 (227/545)
**Variance stabilizing normalization**	465	0.89 (311/351)	0.05 (154/3172)	0.33 (154/465)

### Causes of false positive and false negative markers

There are two possible mechanisms through which false negative (or false positive) markers can occur as a result of array effects and attempted removal of array effects by normalization: one is by introducing bias to the data, and another is by increasing (or decreasing) the variability of the data. In order to examine these two possibilities we looked at scatter plots of the following summary statistics: (1) mean expression differences between two tumor groups for the benchmark data versus that for the test data, in order to look at the level of biases in the test data; and (2) marker-specific standard deviations for the benchmark data versus that for the test data, in order to look at the change of data variability.

**Figure 2:**
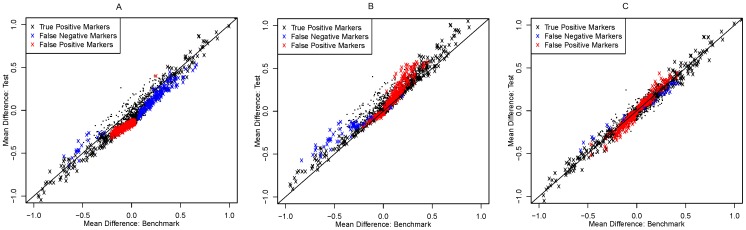
Scatter plot comparing mean differences (ovarian mean – endometrial mean) in the benchmark data and that in the test data for (A) no normalization, (B) median normalization, and (C) quantile normalization. Black “x”: true positive markers. Red “x”: false positive markers. Blue “x”: false negative markers. Black dots: true negative markers.

**Figure 3:**
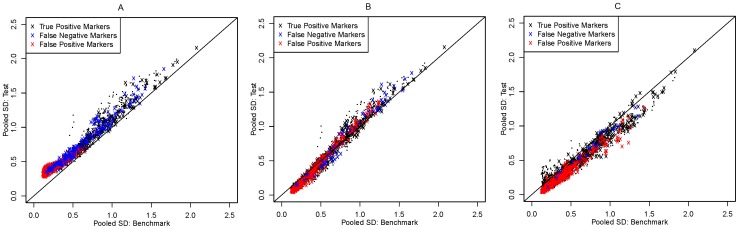
Scatter plot comparing pooled standard deviations in the benchmark data and that in the test data for (A) no normalization, (B) median normalization, and (C) quantile normalization. Black “x”: true positive markers. Red “x”: false positive markers. Blue “x”: false negative markers. Black dots: true negative markers.

Figure 2A and Figure 3A show that array effects led to both a (dominantly negative) bias in mean differences (that is, ovarian mean minus endometrial mean) and an overall increase in variability in the test data. More specifically, the bias primarily shifted the data towards endometrial tumors: it pulled markers whose true mean differences are around zero away from zero, and some markers whose true mean differences are moderately positive close to zero. Most false positive markers had mean differences close to zero in the benchmark and were resulted from the negative biases in mean difference caused by array effects. Most false negative markers had positive mean differences in the benchmark and were resulted from the under-estimated magnitudes of mean difference. The level of increase in data variability is similar for the majority of markers. This increase partly contributed to the occurrence of false negative markers.

We generated similar scatter plots for the test data after median normalization (Figure 2B and Figure 3B). Median normalization corrected the bias in mean difference caused by array effects but with some over-correction. It also decreased the level of data variability but also with a level of over-correction especially for markers whose variability was small in the benchmark. False positive markers after median normalization are primarily caused by over-estimated mean differences and under-estimated standard deviations. Most false positive markers were up-regulated in the benchmark. False negative markers were primarily resulted from under-estimated absolute mean differences, predominantly among markers that are down-regulated in the benchmark.

Similar plots showed that quantile normalization effectively corrected the bias caused by array effects; however, it overly compressed the variability of the data (Figure 2C and Figure 3C). The false positive markers after quantile normalization are primarily caused by under-estimated standard deviations, while the remaining false negative markers are primarily due to under-estimated magnitude of mean differences.

## Conclusions

We utilized a pair of miRNA array datasets on the same set of tumor samples to perform an objective and absolute assessment of normalization performance under genuine data characteristics. Comparing with previous reports on the assessment of normalization methods for miRNA arrays, our approach provides an important alternative evaluation that challenges the critical assumption of the all-gene methods and offers insights on their performance when the assumptions are violated [25]–[27].

In the presence of array effects, normalization and batch adjustment can improve the accuracy of detecting differentially expressed markers. However, the number of false positive markers can still be close to the number of true positive markers as demonstrated in our study.

There is a critical need to develop efficient methods to normalize miRNA array data with both effective bias correction and proper variability reduction so that miRNAs having disease relevance can be identified in an accurate manner.

## Supporting Information

Figure S1 **Scatter plot comparing group means in the randomized dataset.** True positive markers, false negative markers, and false positive markers, as determined in the non-randomized data (without normalization) in comparison with the randomized dataset as the benchmark, are indicated as “x” in black, blue, and red, respectively. True negative markers are indicated as black dots.
(DOCX)

Figure S2 **ROC curves comparing the two-sample t-statistic p-values for the test dataset with (A) no normalization, (B) median normalization, (C) quantile normalization, (D) cyclic loess normalization, and (E) variance stabilizing normalization, with the gold standard (that is, the differential expression status determined by the benchmark dataset).**
(DOCX)

Figure S3 **Comparison of differential expression analysis of the test data before versus after batch adjustment.** Each Venn diagram compares differentially expressed markers identified in the test data after normalization following no BEC (yellow circle) versus those with BEC (using either standardization (green circle) or ComBat (blue circle)).
(DOCX)

Figure S4 **Results of differential expression analysis of the test data before and after batch adjustment, in comparison with the gold standard derived from the benchmark data.** Each Venn diagram compares differentially expressed markers identified in the test data after normalization following no BEC (yellow circle) or BEC (using standardization (green circle) or ComBat (blue circle)) versus those identified in the benchmark data (red circle).
(DOCX)

Table S1 **List of normalization methods examined in our study.**
(DOCX)

## References

[1] Speed T (2003) Statistical Analysis of Gene Expression Microarray Data: CRC Pr I Llc.

[2] Irizarry RA, Bolstad BM, Collin F, Cope LM, Hobbs B, et al (2003a) Summaries of Affymetrix GeneChip probe level data. Nucleic Acids Res 31: e15. 12582260 10.1093/nar/gng015 PMC150247

[3] Qin LX, Satagopan JM (2009) Normalization method for transcriptional studies of heterogeneous samples—simultaneous array normalization and identification of equivalent expression. Stat Appl Genet Mol Biol 8: Article 10. 10.2202/1544-6115.1339 PMC2861326 19222377

[4] Bolstad BM, Irizarry RA, Astrand M, Speed TP (2003) A comparison of normalization methods for high density oligonucleotide array data based on variance and bias. Bioinformatics 19: 185–193. 12538238 10.1093/bioinformatics/19.2.185

[5] Irizarry RA, Hobbs B, Collin F, Beazer-Barclay YD, Antonellis KJ, et al (2003b) Exploration, normalization, and summaries of high density oligonucleotide array probe level data. Biostatistics 4: 249–264. 12925520 10.1093/biostatistics/4.2.249

[6] Ambros V (2004) The functions of animal microRNAs. Nature 431: 350–355. 15372042 10.1038/nature02871

[7] Bartel DP (2004) MicroRNAs: genomics, biogenesis, mechanism, and function. Cell 116: 281–297. 14744438 10.1016/s0092-8674(04)00045-5

[8] He L, Thomson JM, Hemann MT, Hernando-Monge E, Mu D, et al (2005) A microRNA polycistron as a potential human oncogene. Nature 435: 828–833. 15944707 10.1038/nature03552 PMC4599349

[9] Babak T, Zhang W, Morris Q, Blencowe BJ, Hughes TR (2004) Probing microRNAs with microarrays: tissue specificity and functional inference. RNA 10: 1813–1819. 15496526 10.1261/rna.7119904 PMC1370668

[10] Sharma S, Kelly TK, Jones PA (2009) Epigenetics in Cancer. Carcinogenesis. 10.1093/carcin/bgp220 PMC2802667 19752007

[11] Lu J, Getz G, Miska EA, Alvarez-Saavedra E, Lamb J, et al (2005) MicroRNA expression profiles classify human cancers. Nature 435: 834–838. 15944708 10.1038/nature03702

[12] Landgraf P, Rusu M, Sheridan R, Sewer A, Iovino N, et al (2007) A mammalian microRNA expression atlas based on small RNA library sequencing. Cell 129: 1401–1414. 17604727 10.1016/j.cell.2007.04.040 PMC2681231

[13] Esquela-Kerscher A, Slack FJ (2006) Oncomirs - microRNAs with a role in cancer. Nat Rev Cancer 6: 259–269. 16557279 10.1038/nrc1840

[14] Qin LX, Zhou Q, Bogomolniy F, Villafania L, Olvera N, et al (2014) Blocking and Randomization to Improve Molecular Biomarker Discovery. Clin Cancer Res 20: 1–8. 10.1158/1078-0432.CCR-13-3155 PMC4079727 24788100

[15] Kerr MK, Martin M, Churchill GA (2000) Analysis of variance for gene expression microarray data. J Comput Biol 7: 819–837. 11382364 10.1089/10665270050514954

[16] Simon R, Radmacher MD, Dobbin K (2002) Design of studies using DNA microarrays. Genet Epidemiol 23: 21–36. 12112246 10.1002/gepi.202

[17] Tseng GC, Oh MK, Rohlin L, Liao JC, Wong WH (2001) Issues in cDNA microarray analysis: quality filtering, channel normalization, models of variations and assessment of gene effects. Nucleic Acids Res 29: 2549–2557. 11410663 10.1093/nar/29.12.2549 PMC55725

[18] Fan J, Tam P, Vande Woude G, Ren Y (2004) Normalization and analysis of cDNA microarrays using within-array replications applied to neuroblastoma cell response to a cytokine. Proc Natl Acad Sci U S A 101: 1135–1140. 14739336 10.1073/pnas.0307557100 PMC337019

[19] Smyth GK (2004) Linear models and empirical bayes methods for assessing differential expression in microarray experiments. Stat Appl Genet Mol Biol 3: Article3. 16646809 10.2202/1544-6115.1027

[20] Affymetrix Affymetrix Algorithm.

[21] Huber W, von Heydebreck A, Sueltmann H, Poustka A, Vingron M (2003) Parameter estimation for the calibration and variance stabilization of microarray data. Stat Appl Genet Mol Biol 2: Article3. 16646781 10.2202/1544-6115.1008

[22] Johnson WE, Li C, Rabinovic A (2007) Adjusting batch effects in microarray expression data using empirical Bayes methods. Biostatistics 8: 118–127. 16632515 10.1093/biostatistics/kxj037

[23] Chen C, Grennan K, Badner J, Zhang D, Gershon E, et al (2011) Removing batch effects in analysis of expression microarray data: an evaluation of six batch adjustment methods. PLoS One 6: e17238. 21386892 10.1371/journal.pone.0017238 PMC3046121

[24] Pepe MS (2003) The statistical evaluation of medical tests for classification and prediction: Oxford University Press.

[25] Pradervand S, Weber J, Thomas J, Bueno M, Wirapati P, et al (2009) Impact of normalization on miRNA microarray expression profiling. RNA 15: 493–501. 19176604 10.1261/rna.1295509 PMC2657010

[26] Rao Y, Lee Y, Jarjoura D, Ruppert AS, Liu CG, et al (2008) A comparison of normalization techniques for microRNA microarray data. Stat Appl Genet Mol Biol 7: Article22. 18673291 10.2202/1544-6115.1287

[27] Qin LX, Tuschl T, Singer S (2013) An Empirical Evaluation of Normalization Methods for MicroRNA Arrays in a Liposarcoma Study. Cancer Inform 12: 83–101. 23589668 10.4137/CIN.S11384 PMC3615992

