# Supplementary material for: MicroRNA Array Normalization: An Evaluation Using a Randomized Dataset as the Benchmark
Source: PLoS One. 2014 Jun 6;9(6):e98879. doi: 10.1371/journal.pone.0098879 (PMC4048305; doi:10.1371/journal.pone.0098879)
Supplement: Figure S1 — Scatter plot comparing group means in the randomized dataset. True positive markers, false negative markers, and false positive markers, as determined in the non-randomized data (without normalization) in comparison with the randomized dataset as the benchmark, are indicated as “x” in black, blue, and red, respectively. True negative markers are indicated as black dots. (DOCX) [file pone.0098879.s001.docx]

**Supplementary Figure S1.** Scatter plot comparing group means in the randomized dataset. True positive markers, false negative markers, and false positive markers, as determined in the non-randomized data (without normalization) in comparison with the randomized dataset as the benchmark, are indicated as “x” in black, blue, and red, respectively. True negative markers are indicated as black dots.
