# Supplementary material for: MicroRNA Array Normalization: An Evaluation Using a Randomized Dataset as the Benchmark
Source: PLoS One. 2014 Jun 6;9(6):e98879. doi: 10.1371/journal.pone.0098879 (PMC4048305; doi:10.1371/journal.pone.0098879)
Supplement: Figure S2 — ROC curves comparing the two-sample t-statistic p-values for the test dataset with (A) no normalization, (B) median normalization, (C) quantile normalization, (D) cyclic loess normalization, and (E) variance stabilizing normalization, with the gold standard (that is, the differential expression status determined by the benchmark dataset). (DOCX) [file pone.0098879.s002.docx]

**Supplementary Figure S2.** ROC curves comparing the two-sample t-statistic p-values for the test dataset with (A) no normalization, (B) median normalization, (C) quantile normalization, (D) cyclic loess normalization, and (E) variance stabilizing normalization, with the gold standard (that is, the differential expression status determined by the benchmark dataset).
