# Supplementary material for: MicroRNA Array Normalization: An Evaluation Using a Randomized Dataset as the Benchmark
Source: PLoS One. 2014 Jun 6;9(6):e98879. doi: 10.1371/journal.pone.0098879 (PMC4048305; doi:10.1371/journal.pone.0098879)
Supplement: Figure S3 — Comparison of differential expression analysis of the test data before versus after batch adjustment. Each Venn diagram compares differentially expressed markers identified in the test data after normalization following no BEC (yellow circle) versus those with BEC (using either standardization (green circle) or ComBat (blue circle)). (DOCX) [file pone.0098879.s003.docx]

**Supplementary Figure S3.** Comparison of differential expression analysis of the test data before versus after batch adjustment. Each Venn diagram compares differentially expressed markers identified in the test data after normalization following no BEC (yellow circle) versus those with BEC (using either standardization (green circle) or ComBat (blue circle)).
