# Supplementary material for: MicroRNA Array Normalization: An Evaluation Using a Randomized Dataset as the Benchmark
Source: PLoS One. 2014 Jun 6;9(6):e98879. doi: 10.1371/journal.pone.0098879 (PMC4048305; doi:10.1371/journal.pone.0098879)
Supplement: Figure S4 — Results of differential expression analysis of the test data before and after batch adjustment, in comparison with the gold standard derived from the benchmark data. Each Venn diagram compares differentially expressed markers identified in the test data after normalization following no BEC (yellow circle) or BEC (using standardization (green circle) or ComBat (blue circle)) versus those identified in the benchmark data (red circle). (DOCX) [file pone.0098879.s004.docx]

**Supplementary Figure S4.** Results of differential expression analysis of the test data before and after batch adjustment, in comparison with the gold standard derived from the benchmark data. Each Venn diagram compares differentially expressed markers identified in the test data after normalization following no BEC (yellow circle) or BEC (using standardization (green circle) or ComBat (blue circle)) versus those identified in the benchmark data (red circle).
