# Supplementary material for: MicroRNA Array Normalization: An Evaluation Using a Randomized Dataset as the Benchmark
Source: PLoS One. 2014 Jun 6;9(6):e98879. doi: 10.1371/journal.pone.0098879 (PMC4048305; doi:10.1371/journal.pone.0098879)
Supplement: Table S1 — List of normalization methods examined in our study. (DOCX) [file pone.0098879.s005.docx]

**Supplementary Table S1.** List of normalization methods examined in our study.

| **Normalization Method** | **R Package** | **Reference** |
| --- | --- | --- |
| Median Normalization | AFFY | Affymetrix Algorithm |
| Quantile Normalization | AFFY | Bolstad et al. 2003 |
| Cyclic Loess Normalization | AFFY | Bolstad et al. 2003 |
| Variance Stabilizing Normalization | VSN | Huber et al. 2003 |
